# Having a Calling on Board: Effects of Calling on Job Satisfaction and Job Performance Among South Korean Newcomers

**DOI:** 10.3389/fpsyg.2019.01584

**Published:** 2019-07-17

**Authors:** Jiyoung Park, Sinae Kim, Myoungki Lim, Young Woo Sohn

**Affiliations:** Department of Psychology, Yonsei University, Seoul, South Korea

**Keywords:** calling, newcomer, job satisfaction, job performance, job involvement, perceived organizational support

## Abstract

Despite increasing research on calling, how calling functions for those experiencing transition from school to work and how their calling prior to working relates to later well-being and job outcomes has been understudied. The current study explored effects of perceiving a calling on job satisfaction and job performance, as measured at organizational entry and 2 years after organizational entry. Using a time-lagged collection of a sample of South Korean newcomers, the results based on structural equation modeling revealed that perceiving a calling was positively related to supervisor-rated job performance. Job involvement, which was measured 1 year later, fully mediated the relation between perceiving a calling and job satisfaction, but the hypothesized mediating role of job involvement on the link between perceiving a calling and job performance was not supported. We also examined moderating roles of perceived organizational support and perceived person**-**job fit on the relation between perceiving a calling on job involvement and found that perceived organizational support facilitated the effects of perceiving a calling on job involvement. Implications of these findings are discussed.

## Introduction

Researchers have paid increasing attention to a sense of calling as its critical role in understanding attitudes and behaviors related to career and work has been uncovered (Hall and Chandler, 2005; Bunderson and Thompson, 2009; Dik and Duffy, 2009; Wrzesniewski, 2012). Defined as a meaningful job that one uses to help others or contribute to the greater good (Duffy and Dik, 2013), perceiving a calling is positively related to a wide array of job and career outcomes (Wrzesniewski et al., 1997; Duffy et al., 2011). It is generally assumed that people who have a calling experience more work meaningfulness and satisfaction by focusing on the noble goals of their work rather than financial gain (Wrzesniewski et al., 1997; Bunderson and Thompson, 2009). The goals and values of people with a sense of calling enable them to engage in their work (Bunderson and Thompson, 2009), commit to a career (Duffy et al., 2011), continue in the career despite challenges (Dobrow-Riza and Heller, 2015), and perform better (Hall and Chandler, 2005).

To better understand the role of calling across cultures and occupations, researchers have highlighted a need for more diverse research in the calling literature (Duffy and Dik, 2013). An increasing body of research has been conducted with working adults in East Asian countries such as China (Zhang et al., 2015) and South Korea (Park et al., 2016, 2018), but more efforts to investigate calling in diverse contexts are needed to understand how calling functions across cultures (Hunter et al., 2010; Duffy and Dik, 2013). Also, many studies on calling have focused on college students and those who are currently working, and what role calling plays during career transition period has received little attention. Studies based on college students found more than 40% of US college students believed that having a calling was mostly or totally true of them (Duffy and Sedlacek, 2010), and college students who felt the presence of calling were more likely to be decided in their career choices (Duffy and Sedlacek, 2007), prepared better for their career (Shin et al., 2018) and have higher expectations for positive work outcomes (Dik et al., 2008). However, in terms of the callings individuals have prior to working, whether and to what extent these calling result in positive job outcomes and well-being has been understudied.

As a critical period for constructing work identity and work meanings (Ng and Feldman, 2007), the newcomer socialization period can be a beneficial context for examining the antecedents and roles of callings. In particular, this study is based on newcomers who experience their transition from school to work. This transition is a critical and challenging life event, due to the sociological, cultural, and structural differences between the university and the workplace (Kuron et al., 2015). Early work experiences and successful adaptation during the transition lay a foundation for later work identity and work attitudes (Bauer and Green, 1994; Ashforth and Saks, 1996; Bauer et al., 2007). Studies also show that dramatic changes in well-being can occur during the early years of career entry (Boswell et al., 2009), and they tended to be normalized through habituation and desensitization (Ashforth and Kreiner, 2002; Boswell et al., 2009). Focusing on the socialization period, understanding of how perceiving a calling relates to well-being and job performance can be used to inform future studies on research on calling and offer practical implications for leaders and newcomer selection and socialization processes.

The main goal of this study is to examine the effects of newcomers’ calling on their job satisfaction and job performance 2 years after joining the organization. The positive relation between calling and well-being has received consistent support (for a review, Duffy and Dik, 2013), but that of calling and job performance has received relatively less attention and shown mixed findings (Hall and Chandler, 2005; Dobrow, 2013; Park et al., 2016). To explain inconsistent findings between calling and job performance, researchers highlighted a need for longitudinal investigation on effects of calling on job performance (Wrzesniewski, 2012). Given those with callings place more value on their perceived ability than objective ability (Dobrow-Riza and Heller, 2015), using objective job performance such as supervisor-rated job performance would be a critical factor in understanding the relations. This study adds value to calling literature by focusing on effects of calling on supervisor-rated job performance 2 year later. Also, because newcomers typically experience a decrease in their job satisfaction over first few working years (Boswell et al., 2009), understanding a role of presence of calling in well-being for those years offer insights on newcomer literature as well as calling literature. Another aim of this study is to explore how and under what condition perceiving a calling relates to job satisfaction and job performance. We explored a mediating role of job involvement that links from perceiving a calling to job satisfaction and to job performance, and moderating roles of organizational and job factors on the relation between perceiving a calling and job involvement.

### Calling, Job Satisfaction, and Job Performance

Individuals’ perceptions of calling before entering the workforce influence how they think and behave (Bunderson and Thompson, 2009; Hunter et al., 2010; Dobrow-Riza and Heller, 2015). In a large scale of surveys using more than 5,000 college students, >40% of them reported they have a calling (Duffy and Sedlacek, 2010) and students discerned their calling based on diverse sources such as guiding forces, talents, and dedicated efforts (Hunter et al., 2010). College students’ callings had a wide influence on their lives as well as careers (Hunter et al., 2010). Their calling plays a critical role in using and training one’s ability (Hunter et al., 2010) and pursuing one’s career (Bunderson and Thompson, 2009; Dobrow-Riza and Heller, 2015). Researchers note that the callings students had upon entry into a career domain influences later career pursuits by creating psychological conditions under which people focus on their own beliefs about the career domain and by shaping how they think about themselves, their work, and their environment (Dobrow-Riza and Heller, 2015). These findings suggest that the perception of calling upon organizational entry would influence job attitudes and outcomes over time.

For newcomers, reducing uncertainty is a primary goal during the socialization period (Ashford and Black, 1996). Perceiving a calling can reduce employees’ cynicism and sustain their engagement in times of uncertainty by promoting work meaningfulness (Bunderson and Thompson, 2009; Hirschi, 2012) and reaffirming the social influences of their work (Cartwright and Holmes, 2006). People with calling positively frame themselves, their work, and their situation (Dobrow-Riza and Heller, 2015; Schabram and Maitlis, 2017) and newcomer’s positive attitude was found to be effective in increasing job satisfaction (Ashford and Black, 1996). When endorsing a calling, people focus on the ideal and positive aspect of their work (Elangovan et al., 2010) and have a clear goal for their work (Hall and Chandler, 2005). These attitudes, in turn, can contribute to job satisfaction. Having a calling can buffer psychological distress during challenging and uncertain contexts (Schabram and Maitlis, 2017) like newcomer socialization. Although the link between perceiving a calling and job satisfaction has yet to be examined among newcomers, the robust positive relation between perceiving a calling and well-being has received consistent support across diverse occupations (Wrzesniewski et al., 1997; Duffy et al., 2012; Conway et al., 2015). Based on these findings, we expected that perceiving a calling upon organizational entry would positively relate to job satisfaction 2 years later.

*Hypothesis 1*: Perceiving a calling is positively related to job satisfaction.

In predicting newcomers’ performance and adaptive behaviors, a significant predictor is newcomers’ proactive behavior at work (Ashford and Black, 1996). Among diverse proactive tactics, such as feedback seeking and networking, self-focused proactive strategies such as positive reframing were positively related to self-rated job performance (Ashford and Black, 1996). An implicit assumption about calling is that people are called to a specific way of work that motivates a course of action to achieve one’s goals (Elangovan et al., 2010). Scholars note that people with a sense of calling demonstrate high job performance because they establish clear goals and exert greater efforts to accomplish them (Hall and Chandler, 2005; Wrzesniewski, 2012). Individuals with callings are more engaged in their work (Bunderson and Thompson, 2009) and confront situations more proactively not because of situational affordance, but due to their mindset (Schabram and Maitlis, 2017). Thus, we hypothesized that perception of a calling upon organizational entry would be positively related to job performance.

*Hypothesis 2*: Perceiving a calling is positively related to job performance.

### Job Involvement as a Mediator

Job involvement is defined as a cognitive belief of psychological identification with one’s job (Kanungo, 1982). Job involvement represents the strength of one’s attachment to a work (Kanungo, 1982). Among diverse occupations of US workers, viewing work as a calling was positively related to one’s belief that work is one of the most important things in their life (Wrzesniewski et al., 1997). Qualitative research by Bunderson and Thompson (2009) on zookeepers found that a sense of calling enables them to maintain a high level of job involvement by infusing even trivial tasks with transcendent meaning and significance, and the authors assert that zookeepers’ callings provide a compelling basis for identification with the occupation.

Although a causal relation between perceiving a calling and job involvement has received little attention from researchers, we expect that perceiving a calling would promote one’s attachment to work and psychological identification with one’s work. Hackman and Oldham (1975) suggest that intrinsic perceptions of job characteristics, such as work meaningfulness, increase job involvement. Those with callings show increased dedication (Hunter et al., 2010; Praskova et al., 2014) and the continuous efforts, which result in increased work meaningfulness (Hirschi, 2012) and job involvement (Brown, 1996). Also, people with callings seek to live out their calling (Elangovan et al., 2010) and have a strong desire to fulfill it by engaging in job crafting activities (Berg et al., 2010). Such job crafting behaviors longitudinally contribute to work meaningfulness (Tims et al., 2016), which leads to increased job involvement (Hackman and Oldham, 1975). Findings that show that job involvement is predicted by positive traits such as self-esteem and a clear sense of self (Brown, 1996), which are closely related to calling (Hall and Chandler, 2005), also support the positive link between perceiving a calling and job involvement. Based on these findings, we hypothesized,

*Hypothesis 3*: Perceiving a calling is positively related to job involvement.

Increased job involvement may be one route by which perceiving a calling relates to job satisfaction and job performance. Scholars have noted that fostering identification with occupation serves a means by which callings create attitudes toward jobs (Bunderson and Thompson, 2009). For example, a sense of a calling has a strong positive relation with occupational identification (Bunderson and Thompson, 2009), and occupational identification and occupational identity mediated the relation between calling and well-being at work (Bunderson and Thompson, 2009; Hirschi, 2012). According to a theoretical model of job involvement (Brown, 1996), those who view themselves as competent are more likely to seek challenges to improve personal growth at work, and the greater growth needs motivate them to engage in job activities that result in increased job satisfaction (Brown, 1996). Other findings suggest that the positive self-views (Hirschi, 2012; Dobrow-Riza and Heller, 2015) and strong growth initiative of those with callings (Bott and Duffy, 2015; Schabram and Maitlis, 2017) render these individuals to feel more satisfied with their jobs via increased job involvement.

Also, the findings that career commitment, a construct largely overlapped with job involvement (Brown, 1996), fully mediates the relation between perceiving a calling and job satisfaction (Duffy et al., 2011) also lends support to the mediating role of job involvement on calling and job satisfaction. An article that suggests a theoretical model of work as a calling, the work as calling theory (WCT), underlines that perceiving a calling predicts greater career commitment, which in turn predicts job outcomes (Duffy et al., 2018). As the first formal attempt to establish a theoretical model of calling, the WCT argues that the level of commitment in career or work would mediate the link of perceiving a calling and positive job outcomes such as job satisfaction and job performance (Duffy et al., 2018).

Given that job involvement is a primary factor in predicting organizational effectiveness (Pfeffer, 1994) and individual motivation (Hackman and Lawler, 1971), the relation between perceiving a calling and job performance can be explained by job involvement. Scholars argue that the influences of perceiving a calling on job performance can be accounted for by the continued effort and motivation of those with callings (Hall and Chandler, 2005; Wrzesniewski, 2012), as these individuals overcome obstacles at work by enhancing their skills and accumulating knowledge (Praskova et al., 2014; Schabram and Maitlis, 2017). Those with callings are likely to be immersed in their work (Bunderson and Thompson, 2009) and the motivational processes influence job performance (Diefendorff et al., 2002). Although a mediating role of job involvement on the relation between perceiving a calling and job performance has yet to be examined, job involvement serves as a mediating mechanism that connects individual’s traits and job outcomes (Brown, 1996). Traits that are predisposed to job involvement include internal motivation (Conway et al., 2015) and the strength of growth need (Bott and Duffy, 2015; Schabram and Maitlis, 2017), which are possessed by those with high levels of callings. Based on these findings, we hypothesized that job involvement would be a route by which perceiving a calling relate to job satisfaction and job performance.

*Hypothesis 4*: Job involvement mediates the relation between perceiving a calling and job satisfaction.
*Hypothesis 5*: Job involvement mediates the relation between perceiving a calling and job performance.

### The Moderating Roles of Perceived Organizational Support and Perceived Person-Job Fit

The effects of perceiving a calling on job involvement can be conditional depending on perceptions about job and organization; also, individual and contextual factors play an important role in understanding the effects of callings (Hall and Chandler, 2005; Duffy et al., 2017). In explaining the interactive effects, Duffy et al. (2017) suggest that calling can be understood through motivational theories such as Self-Determination Theory (Deci and Ryan, 2000). According to this theory, the effects of goal pursuit and attainment concern the degree to which people are able to satisfy their needs for competence, relatedness, and autonomy (Deci and Ryan, 2000). The psychological needs for different types of motivation vary depending on individual and contextual factors and human needs specify the necessary conditions for well-being and adaptive behaviors (Deci and Ryan, 2000). In framing calling from a goal-setting perspective (Hall and Chandler, 2005; Praskova et al., 2014), researchers found that young adults engage in more goal-oriented cognition, activities, and career strategies (Praskova et al., 2014). Focusing on the motivation concept from Self-Determination Theory, a study (Duffy et al., 2017) found that calling motivation strengthened the relation between perceiving a calling and well-being. Qualitative research has also found that the effects of having a calling on job attitudes and job behaviors are facilitated and constrained by individual factors such as goal orientation and organizational factors such as developmental relationships (Schabram and Maitlis, 2017). These findings suggest that people with callings are oriented toward accomplishing goals and individual and social factors that satisfy the basic psychological needs can facilitate the effects of perceiving a calling on job perceptions, attitudes, and behaviors.

Perceived organizational support refers to employees’ general perceptions of being valued and cared about by their organization, and is based on quality social interactions at work (Eisenberger et al., 1990). Self-Determination Theory suggests that supportive environment can be an important condition to satisfy basic psychological needs (Deci and Ryan, 2000). When organization was perceived as supportive, employees feel free to express themselves, feel more connected and perceived their roles as challenging, thus their needs for competence, relatedness, and autonomy are likely to be fulfilled (Deci and Ryan, 2000; Greguras and Diefendorff, 2009). People with callings have a strong desire to pursue their goals (Hall and Chandler, 2005), in an environment that satisfies basic psychological needs, they are more likely to involve in their job. For example, people with calling view their social relations as a special bonding and they exert more effort based on the strong relatedness (Bunderson and Thompson, 2009). However, when individuals did not feel they received enough support from the organization and feel threatened by depleting interpersonal relations, people with callings experienced a sense of defeat in pursuing their callings and guard themselves from their work (Schabram and Maitlis, 2017). The moderating role of perceived organizational support on the link between perceiving a calling on work commitment such as job involvement is also supported by the WCT (Duffy et al., 2018). The theoretical model suggests that those who perceive a calling and who experience high levels of organizational support are likely to feel their work environment fit well and experience positive outcomes (Duffy et al., 2018). The social capital resulting from supportive social environment may facilitate beneficial effects of perceiving a calling while the lack of organizational support is likely to be perceived as a barrier to live out their calling and to perform positive behaviors at work (Duffy et al., 2018). Based on these theoretical and empirical findings, we hypothesized that perceived organizational support strengthen the positive effects of perceiving a calling on job involvement.

Jobs that provide opportunities to express an individuals’ values and beliefs (Shamir, 1991) are more likely to facilitate the effects of calling on job involvement. When employee perceives their abilities and skills that match the requirements of the job, they feel their need for competence satisfied (Greguras and Diefendorff, 2009). People seek work that can express their authentic self (Shamir, 1991), and feel authentic and competent in situations that allow for value attainment (Schlegel et al., 2009). When people with callings perceive high levels of person**-**job fit, they are likely to find more meaningfulness in their work and become more involved in their work (May et al., 2004). However, when their job does not fit with their core values, people with callings experience regret and distress due to difficulties in pursuing their callings (Berg et al., 2010). Thus, we hypothesized that those with calling would be more involved in their job when they perceive higher person**-**job fit.

*Hypothesis 6*: Perceived organizational support moderates the relation between perceiving a calling and job involvement, such that those with high perceived organizational support show stronger relations than those with low perceived organizational support.
*Hypothesis 7*: Perceived person**-**job fit moderates the relation between perceiving a calling and job involvement, such that those with high perceived person**-**job fit show stronger relations than those with low perceived person**-**job fit.

## Methods

### Participants and Procedure

Our sample consisted of new employees of a company headquartered in South Korea. The company’s concerns ranged from electronics to financial investment. Surveys were administered at three time points during new employees’ first two working years: during the first week of the orientation program (Time 1; T1); 1 year (Time 2; T2); and 2 years (Time 3; T3) after organizational entry. Measures at T1 were obtained during the first week of a newcomer’s orientation, before they had met their supervisor, been assigned to a team, or begun undertaking tasks. An HR manager at the orientation informed employees that the survey was voluntary and irrelevant to the organization, and posted the survey link on an Internet community board used for the 3-week orientation program. Email addresses were collected when new employees signed and submitted their informed consent form. Employees were assigned to a team after completing the orientation program. At T2 and T3, survey links were sent via email by one of the authors, who is employed by a research institution affiliated with the company.

The T1 survey collected data on the newcomer’s calling along with a control variable, core self-evaluations, and demographic information. The T2 survey collected data on job involvement, perceived person**-**job fit, and perceived organizational support and at T3, newcomers’ job satisfaction was measured via survey and task performance data were obtained from the company. All surveys were administered in Korean, and all measures were translated into and validated in Korean.

Five hundred thirty participants completed the survey at T1, 246 at T2, and 145 at T3. We sent emails to all participants who did not complete surveys but had consented to receive survey emails. Two hundred twenty-one completed both T1 and T2 surveys; and 145 completed all three surveys. Of the participants who responded to the T1 survey, all were Korean, 74.3% were male, and the average age was 25.32 years (*SD* = 1.83). Educational level was measured using four categories – high school graduate, 2-year college or technical college graduate, 4-year university graduate, and graduate school – and the category was coded on a scale from1 to 4. Most participants (90.6%) had a least 4-year university degree.

In examining the hypotheses, we controlled for the effects of core self-evaluations. Core self-evaluations represent the basic and fundamental beliefs individuals have about themselves and their functioning in the world (Judge et al., 2003). Core self-evaluations consist of four personality traits: self-esteem, generalized self-efficacy, locus of control, and emotional stability (Judge et al., 2003). Core self-evaluations have been found to be a significant predictor of job satisfaction and job performance across diverse occupations of working adults (Judge and Bono, 2001; Judge et al., 2003). Also, on average, a positive moderate correlation between perceiving a calling and core self-evaluations was found among diverse groups of samples (Duffy et al., 2012; Hirschi and Herrmann, 2013). When investigating the relations between calling, job satisfaction, and job performance, it is important to account for the effects of a basic personality trait (Hirschi and Herrmann, 2013). Controlling for the effects of core self-evaluations on job satisfaction and job performance yields stronger inferences regarding the hypothesized relations and more precise understanding of the relations.

### Instruments

#### Calling

We assessed the extent to which employees perceived a sense of calling using the 12-item Presence of Calling scale from the Calling and Vocation Questionnaire (CVQ; Dik et al., 2012). A sample item includes “I see my career as a path to purpose in life.” The measure was assessed on a five-point Likert scale ranging from 1 (*Not at all true of me*) to 5 (*Absolutely true of m*e). In the current study, the estimated internal consistency reliability of the scale scores was α = 0.70.

#### Job Involvement

We assessed job involvement using the Job Involvement Questionnaire developed by Kanungo (1982). The 10-item scale measures the extent to which individuals identify psychologically with their jobs. Sample items include “I am very much involved personally in my job” and “Usually I feel detached from my job” (reverse-coded). The measure was assessed on a five-point Likert scale ranging from 1 (*Strongly disagree*) to 5 (*Strongly agree*). In the current study, the estimated internal consistency reliability of the scale scores was α = 0.84.

#### Perceived Organizational Support

To measure the extent to which employees perceive their organization as valuing their well-being and contributions, we used the Survey of Perceived Organizational Support (Eisenberger et al., 1986). Of the 17 items, we used 9 items with highest loadings; this is in line with past research (Eisenberger et al., 1990). Sample items are “The organization strongly considers my goals and values” and “The organization really cares about my well-being.” The measure was assessed on a five-point Likert scale ranging from 1 (*Strongly disagree*) to 5 (*Strongly agree*). In the current study, the estimated internal consistency reliability of the scale scores was α = 0.90.

#### Perceived Person-Job Fit

We assessed employee perceptions of job fit in terms of skills, abilities, and personalities using five items developed by Lauver and Kristof-Brown (2001). A sample item is “There is a good match between the requirements of this job and my skills.” Respondents indicated their level of agreement with each statement on a seven-point Likert scale ranging from 1 (*Strongly disagree*) to 7 (*Strongly agree*). In the current study, the estimated internal consistency reliability of the scale scores was α = 0.91.

#### Job Satisfaction

Job satisfaction was measured with the six items developed by Hackman and Oldham (1975), using one item on general job satisfaction and five items on specific aspects of jobs, such as job security and opportunity for personal development. A sample item is “In general, I am satisfied with my job.” Participants responded to each item using a five-point Likert scale ranging from 1 (*Strongly disagree*) to 5 (S*trongly agree*). In the current study, the estimated internal consistency reliability of the scale scores was α = 0.91.

#### Job Performance

The company had implemented a new employee guidance and observation system for the first year, during which performance was not evaluated. The company evaluated new employees’ first performance 2 years after joining the company, and these results were used in the study. The company employed a forced distribution ranking system that requires raters to evaluate employees by placing them into predetermined percentage groups (Schleicher et al., 2009). The forced distribution ranking system approach is a relative rating technique in which employees’ performance ratings are evaluated by comparing coworkers’ performance (Schleicher et al., 2009; Giumetti et al., 2015). The company rated employees using five percentage levels: 10% for Excellent, 25% for Very Good, 55% for Good, 10% for Needs Improvement, and Unsatisfactory. The last level, Unsatisfactory, is applied only when necessary. Performance data in this study include all five levels, with the following percentage for each: 0.8% Excellent, 14.5% Very Good, 73.8% Good, 8.3% Needs Improvement, and 0.7% Unsatisfactory. Although the performance appraisal is an ordinal variable, five or more categories can be converted to continuous variables (Rhemtulla et al., 2012). Thus, we converted the data into a five-point Likert scale ranging from 1 (*Needs Improvement*) to 5 (*Excellent*).

#### Core Self-Evaluations

Participants measured the extent to which they appraised their worthiness and capabilities in their organization. Twelve items from the Core Self-Evaluation Scale (CSES; Judge et al., 2003) were used. The items were rated on a five-point Likert scale ranging from 1 (*Strongly disagree*) to 5 (*Strongly agree*). A sample example includes “I determine what will happen in my life.” In the current study, the estimated internal consistency reliability of the scale scores was α = 0.83.

## Results

### Hypotheses Testing

First, we performed confirmatory analysis (CFA) with maximum likelihood estimation using AMOS 18. As fit indices, three fit indices were used in addition to the Chi-square test – the Tucker–Lewis index (TLI), the comparative fit index (CFI), and the root-mean-square error of approximation (RMSEA) with the following guidelines: Values of 0.90 can be defined as acceptable to assess the fit of TLI and CFI (Hu and Bentler, 1999). Values of 0.5 for the RMSEA indicate close fit, values in the vicinity of 0.08 indicate fair fit, and values of 0.10 and larger indicate poor fit (Browne and Cudeck, 1993).

Parcels were created using a method proposed by Little et al. (2002). For a calling, the three subscales were used as observed indictors. For job involvement, job satisfaction, and perceived organizational support, which have more than five observed indicators, parcels were created with a balancing assignment (Little et al., 2002). Two 3-item parcels and one 4-item parcels for job involvement, two 3-item parcels for job satisfaction and three 3-item parcels for perceived organizational support were created according to the size of the factor loading. Although perceived person**-**job fit has five observed indicators, to create an interaction term using a matched-pair strategy (Marsh et al., 2004), we created two 2-item parcels and one observed indicator that match with the three parcels of calling. For job performance, one observed variable was used.

We used structural equation modeling to test our hypotheses. Maximum likelihood was used to estimate missing data. As with most longitudinal data, some participants were unavailable at one or more time points. We used full-information maximum-likelihood (FIML) estimation, which uses full information from all observations (Yuan and Bentler, 2000) but yields relatively unbiased results (Arbuckle, 1996). FIML has been found to be efficient for incomplete data (Schafer and Olsen, 1998), and is highly recommended for structural equation modeling analysis using incomplete data (Arbuckle, 1996). As normality assumptions are important for FIML estimation, we investigated the normality of each variable whether skewness had an absolute value of 2.0 or larger and standardized kurtosis had an absolute value of 7.0 or larger (West et al., 1995). All study variables were well-suited to the guidelines. We also conducted an attrition analysis by comparing those who completed surveys at all time points (*N* = 145) and those who completed the T1 survey only (*N* = 385). No differences in levels of initial calling, core self-evaluations, age, gender ratio, or education level were found. The results suggest that, overall, attrition was not systematic with regard to study variables.

To examine moderating effects, we followed a two-stage maximum-likelihood approach described by Ping (1996). In stage 1, we obtained estimates of factor loadings, factor covariances, and error variances from the structural equation model without interaction terms. In stage 2, we added interaction terms. To create latent construct interactions, we multiplied standardized indicators of independent and moderating variables (Ping, 1996; Marsh et al., 2004). Using a matched-pair strategy, we created interaction terms with three latent variables.

Table 1 shows the means, standard deviations, reliabilities, and correlations among the study variables. Calling positively correlated to supervisor-rated job performance (*r* = 0.42, *p* < 0.01), but did not correlated to job satisfaction (*r* = −0.01, *ns*). The correlation between calling and job involvement was also positive (*r* = 0.21, *p* < 0.01). In regard to moderators, calling related neither to perceived organizational support (*r* = 0.09, *ns*) nor perceived person**-**job fit (*r* = 0.06, *ns*). Job involvement had positive correlations with both perceived organizational support (*r* = 0.23, *p* < 0.01) and perceived person**-**job fit (*r* = 0.21, *p* < 0.01). Because education level was positively related to job performance, we controlled for it in the structural model analyses.

**TABLE 1:** Descriptive statistics and correlations for study variables.

**Variable**	**Mean**	**SD**	**1**	**2**	**3**	**4**	**5**	**6**	**7**	**8**	**9**
1. Gender (T1)	1.26	0.44	−								
2. Age (T1)	25.32	1.83	−0.50^∗∗^	−							
3. Educational level (T1)	3.08	0.31	0.04	0.30^∗∗^	−						
4. Calling (T1)	3.58	0.47	−0.09^∗∗^	0.05^∗∗^	−0.04^*^	−					
5. Core self-evaluations (T1)	3.99	0.41	−0.20^∗∗^	0.02^∗∗^	0.03^*^	0.20^∗∗^	−				
6. Job involvement (T2)	3.27	0.59	0.05^∗∗^	−0.06^∗∗^	0.11^*^	0.21^∗∗^	0.05^∗∗^	−			
7. Perceived person-job fit (T2)	4.72	1.09	−0.14^∗∗^	0.03^∗∗^	−0.02^*^	0.09^∗∗^	0.21^∗∗^	0.45^∗∗^	−		
8. Perceived organizational support (T2)	3.38	0.66	−0.14^∗∗^	0.03^∗∗^	0.01^*^	0.06^∗∗^	0.23^∗∗^	0.53^∗∗^	0.53^∗∗^	−	
9. Job satisfaction (T3)	3.27	0.82	−0.04^∗∗^	−0.02^∗∗^	−0.04^*^	−0.01^∗∗^	0.13^∗∗^	0.34^∗∗^	0.30^∗∗^	0.49^*^	−
10. Job performance (T3)	3.10	0.60	−0.05^∗∗^	0.17^∗∗^	0.20^*^	0.24^∗∗^	0.06^∗∗^	0.11^∗∗^	−0.02^∗∗^	−0.05^*^	0.01

*N* = 145–530. Gender 1, male; T1, Time 1; T2, Time 2; T3, Time 3. ^*^p < 0.05, ^∗∗^*p* < 0.01.

Before evaluating the structural models, we examined the fit indices of a measurement model and evaluated how well indicators loaded onto their factors. The measurement model had good fit to the data, χ^2^(*df* = 150) = 280.30, *p* < 0.01, CFI = 0.96, TLI = 0.95, and RMSEA = 0.04. Also, all indicators were loaded onto their respective latent variables at values of 0.71 or higher, showing strong relations between observed indicators and latent factors.

We then assessed the fit of our hypothesized model to the data. We specified a model in which perceiving a calling predicts job involvement, which in turn predicts job satisfaction and job performance. We specified a partial mediation model that included these relationships, together with direct effects of the independent variables on the dependent variables when controlling for core self-valuations and education level. This model showed a good fit, χ^2^(*df* = 71) = 139.62, *p* < 0.01; CFI = 0.97, TLI = 0.95, RMSEA = 0.04. Perceiving a calling was positively related to job involvement (β = 0.25, *p* < 0.01). The path from job involvement to job satisfaction was positive and significant (β = 0.42, *p* < 0.01). However, there were no statistically significant links from job involvement to job performance (β = 0.08, *ns*), and from perceiving a calling to job satisfaction (β = −0.12, *ns*). Due to a non-significant relation between job involvement and job performance, the mediating role of job involvement was not examined; thus, Hypothesis 5 was rejected.

To improve model fit indices, we excluded all nonsignificant paths that include a path from calling to job satisfaction and a path from job involvement to job performance. This model showed similar fit indices, χ^2^(*df* = 73) = 141.55, *p* < 0.01; CFI = 0.97, TLI = 0.95, RMSEA = 0.04, but the difference in Chi-square between the two model was significant, *p* < 0.05. Thus, we chose this model as a final model without interaction terms. Consistent with Hypothesis 3, the path from calling to job involvement was positive (β = 0.24, *p* < 0.01) and the link from job involvement to job satisfaction was significant and positive (β = 0.39, *p* < 0.01). The path from calling to job performance was significant (β = 0.27, *p* < 0.01), supporting Hypothesis 2.

We tested the significance of indirect effects using a Monte Carlo approach that constructs the appropriate confidence intervals (CIs) (Preacher and Selig, 2012). We entered standardized coefficients and standard errors on the relation between calling and job involvement (β = 0.24, *SE* = 0.09) and the relation between job involvement and job satisfaction (β = 0.47, *SE* = 0.11) into a web-based Monte Carlo calculator to compute a 95% CI based on 20,000 simulated draws from the distributions for the parameters (Preacher and Selig, 2012). The relationship between perceiving a calling and job satisfaction was fully mediated by job involvement (95% CI 0.02, 0.22), which supports Hypothesis 4.

### Testing Moderation

All moderated effects were tested using the procedure recommended by Ping (1996). However, it should be noted that not all of the possible observed variable cross-product terms were used when constructing the latent interaction term. Jöreskog and Yang (1996) demonstrated that it is sufficient to construct the latent interaction terms using a matched-pair approach. To create an interaction term, we centered the independent variable (i.e., calling) and moderators (i.e., perceived organizational support, perceived person**-**job fit). Then, we multiplied standardized indicators of independent and moderating variables to create latent construct variables. We obtained estimates of factor loadings, factor covariances, and error variances from the fully mediated model without interaction terms. Then, we added two interaction terms along with the two moderators. When the interaction terms are included in the model, the focus is solely on the significance of the estimated effects of the latent interaction constructs on job involvement because the interaction is orthogonal to the main effects of the constructs (Marsh et al., 2004; Little et al., 2002).

**FIGURE 1:**
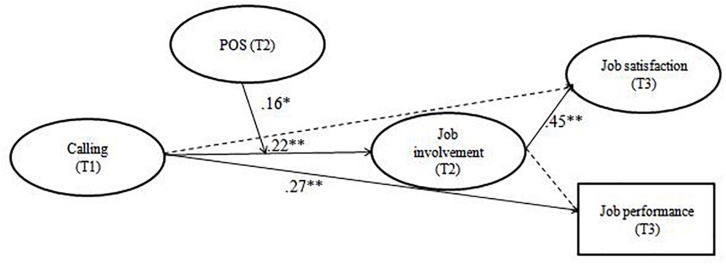
Final model with calling predicting job satisfaction and job performance via job involvement and a moderating effect of perceived organizational support (POS) on the relation between calling and job involvement. Control variables are not shown for simplicity. T1 = Time 1, T2 = Time 2, T3 = Time 3. ^*^p < 0.05, ^∗∗^*p* < 0.01.

We added interaction terms for perceived organizational support and perceived person**-**job fit. The model showed an acceptable fit, χ^2^(*df* = 287) = 689.64, *p* < 0.01, CFI = 0.90, TLI = 0.87, RMSEA = 0.05. The interaction of calling and perceived organizational support on job involvement was significant (β = 0.26, *p* < 0.01), while the interaction of calling and person**-**job fit was not significant (β = −0.14, *ns*), rejecting Hypothesis 7. The model without the person**-**job fit interaction showed better fit indices, χ^2^(*df* = 164) = 340.11, *p* < 0.01, CFI = 0.94, TLI = 0.93, RMSEA = 0.04. Path estimates for this model were shown in Figure 1. Consistent with Hypothesis 6, the interactive effect of perceived organizational support and calling on job involvement was significant (β = 0.16, *p* < 0.05).

**FIGURE 2:**
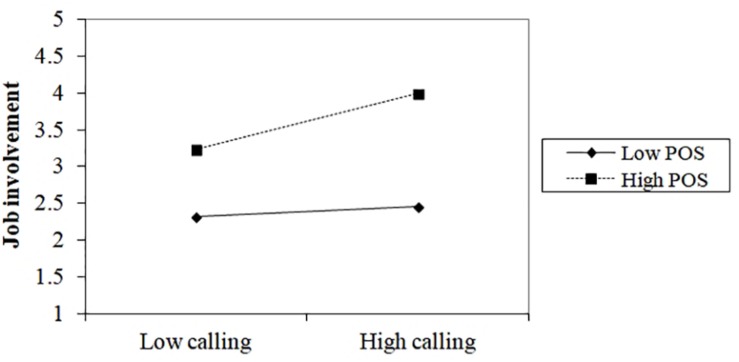
Interaction of perceiving a calling and perceived organizational support (POS) on job involvement.

To interpret the nature of the interaction, we plotted the relations using a procedure recommended by Aiken et al. (1991). We plotted the relation between perceiving a calling and job involvement that corresponds to the low (one standard deviation above the mean) and the high (one standardized deviation below the mean) values of perceived organizational support (Figure 2). Our findings show that, when newcomers perceived high levels of organizational support, their calling had a positive influence on job involvement (β = 0.37, *t* = 3.77, *p* < 0.05). However, when newcomers perceived low levels of organizational support, perceiving a calling was not statistically related to job involvement (β = 0.06, *t* = 0.56, *ns*). This suggests that under high levels of perceived organizational support, calling have a greater positive influence on job involvement than in situations in which they received little support from their organization.

## Discussion

Drawing from previous research on calling, this study examined how and through which mechanism the effects of perceiving a calling relate to job satisfaction and job performance among South Korean newcomers. Controlling for the effects of core self-evaluations and education level, the structural model demonstrated that perceiving a calling at organizational entry was positively related to job satisfaction and job performance 2 years after newcomers joined the organization. Also, job involvement measured 1 year after entry mediated the relation between perceiving a calling and job satisfaction, and perceived organizational support strengthened the positive effects of perceiving a calling on job involvement. In particular, this study shows how perceiving a calling upon entry to workforce relates to individual and organizational well-being over time. Although research on calling has uncovered the benefits of college students’ career calling on their career and life attitudes, how their calling relates to well-being and job outcomes during socialization period has been understudied. This study reveals that those experiencing transition from school to work benefit from perceiving a calling in terms of their well-being and job performance.

We found that the relation between perceiving a calling and job satisfaction was fully explained by the level of job involvement after controlling for the effects of core self-evaluations and education level. Although calling cannot be defined as the strength of work attachment (Wrzesniewski, 2012), our results indicate that those with callings are deeply involved in their work and are likely to feel satisfied with their job through involvement in their job. Job involvement reflects the extent to which individuals identify with their work, based on their investment of time and energy in current roles (Brown and Leigh, 1996). Consistent with prior research on calling (Bunderson and Thompson, 2009; Schabram and Maitlis, 2017), it is likely that employees who view their work as a calling put more effort and investment into their careers, which, in turn, increases their job satisfaction. Existing research on the relation between perceiving a calling and well-being such that perceiving a calling does not necessarily lead to job satisfaction unless people are committed to their careers (Duffy et al., 2011) or live out their callings (Berg et al., 2010; Duffy et al., 2013). The results of this study reveal that job involvement is also a critical factor that links the relation between calling and job satisfaction. Also, the finding that perceiving a calling prior to working engenders beneficial effects on well-being through their job involvement offers insights for the newcomer socialization literature. The results are in line with the findings that attitudes toward career can have significant effects during job search and its outcomes (Guan et al., 2013). As job satisfaction and commitment generally decline over the first few years (Boswell et al., 2009), having a sense of calling and maintaining job involvement can be beneficial in terms of sustaining well-being during newcomer socialization period.

Another contribution of this study is its exploration of the effects of perceiving callings upon career entry on job performance. The degree to which newcomers perceived a calling even before being assigned to a team and task was positively related to supervisor-rated performance 2 years later. This finding is consistent with the research that demonstrates associations between calling and a stronger sense of duty and sacrifice (Bunderson and Thompson, 2009) and fewer missed days of work (Wrzesniewski et al., 1997). Although some research has found that having a calling is not related to objective ability (Dobrow-Riza and Heller, 2015) or objective job performance (Park et al., 2016), the finding of this study supports that perceiving a calling eventually leads to objective success (Hall and Chandler, 2005; Wrzesniewski, 2012; Schabram and Maitlis, 2017). Given prior research used cross-sectional data of calling and job performance (Park et al., 2016), this study offers insights on the relation between calling and job performance such that it may take relatively long time to observe a positive association between having a calling and objective job performance after one begins a professional career. More longitudinal research is needed to bolster the finding and understand the relation between calling and job performance.

Despite the positive relation between calling and performance, in this study, why perceiving calling results in better job performance remains unknown. Due to the non-significant relation between job involvement and job performance, the mediating role of job involvement on the relation was not supported. The effects of job involvement on job performance have received mixed findings (Brown, 1996). While some research has found that job involvement predicts supervisor-rated job performance (Diefendorff et al., 2002), a meta-analysis concluded that there is little support for a positive relation between job involvement and job performance (Brown, 1996). Researchers suggest that, when exploring the relation between job involvement and job performance, controlling for the effects of work centrality is necessary to reflect the true relation between the two (Paullay et al., 1994; Diefendorff et al., 2002). According to these researchers, the degree to which individuals are preoccupied with their work predicts job performance rather than the relative importance of work compared with other aspects of life (Paullay et al., 1994; Diefendorff et al., 2002). In this study, we did not control for work centrality, and this may be one reason for the non-significant relation between job involvement and job performance.

In regard to moderators, we found an interactive effect of calling and perceived organizational support on job involvement; the results speak directly to the key role of social membership and supportive environment in facilitating effects of perceiving a calling (Cardador and Caza, 2012). This is also in line with prior findings that amateur musicians’ initial calling upon career entry had a positive relationship with their perceived social comfort in the career domain (Dobrow, 2013). We note that cultural differences may partly account for the effects of perceived organizational support on calling, given that social ties and interconnectedness are decisive factors in predicting positive attitudes and behaviors in East Asian culture (Kitayama et al., 2000). Also, this study was conducted during socialization period, HR practices, and perceptions of HR policies may influence the relations of the study variables. For example, employees perceive HR practices and policies as a part of wider domain of perceived organizational support and the attractive and fair policies can promote employee’s motivation (Barattucci et al., 2017). A more nuanced approach using different types of fairness or justice perceptions in HR practices can help clarify what aspects of perceived organizational support affect the relation between newcomer’s calling and job involvement.

The hypothesized interactive effect of calling and perceived person**-**job fit on job involvement was not supported in this study. It seems that, once employees view their work as a calling, their perspective toward a work causes involvement in their job regardless of their perceived fit with a job. Interestingly, most studies on calling and person**-**job fit have failed to show hypothesized conditional or distinctive effects of person**-**job fit among those with calling (Hirschi, 2012; Duffy et al., 2014). Scholars suggest that perceived person**-**job fit may function as a process for discerning a sense of calling, and it may not impact the consequences of perceiving a calling, as long as people live out their callings (Duffy et al., 2014). Also, when people view their work as a calling, they are involved in their job by training themselves rather than questioning their fit with work (Schabram and Maitlis, 2017). According to a qualitative study on animal shelter workers (Schabram and Maitlis, 2017), those who have a sense of calling despite challenges did not necessarily consider themselves to have a unique fit for their work. Rather, they focused on how to learn new tasks and build their capacities in response to the challenges (Schabram and Maitlis, 2017).

The hypothesized model suggests perceiving calling results in higher job satisfaction and job performance through job involvement and the psychological mechanism is moderated by perceptions about organization. Given scholars suggest that living a calling serves as a mediator when explaining the relation between perceiving a calling to life and job outcomes (Duffy and Dik, 2013), job involvement can be one route by which perceiving a calling relates to living a calling. Also, in line with prior research (Duffy et al., 2018), the findings support that perceived organization support is a critical factor in understanding how perceiving a calling relates to job outcomes. Although perceived organizational support is based on quality interaction at work, perceptions about organization had associations with all of the basic psychological needs for competence, relatedness, and autonomy (Greguras and Diefendorff, 2009). Based on Self-Determination Theory, investigation on what specific aspects of perceived organizational support strengthen the effects of perceiving a calling on job involvement can contribute to better understanding on calling.

The results of the present study have some practical implications. First, our findings that newcomers’ calling upon organizational entry was positively related to job involvement, job satisfaction, and job performance suggest that human resource (HR) managers should take applicants’ calling into consideration to warrant newcomer well-being and promote performance. Given that newcomers generally experience a decline in job satisfaction for first few working years (Boswell et al., 2009), selecting newcomers with a high sense of calling or developing a sense of calling can be a way to promote their well-being and job performance. In particular, job satisfaction is a critical indicator of predicting turnover, which is peaked during first few years upon organizational entry or job change (Judge and Kammeyer-Mueller, 2012), and job satisfaction had more direct effects on turnover during newcomer socialization period (Dickter et al., 1996). Because of additional HR costs, such as recruitment, selection, on-boarding training costs, maintaining retention of newcomers and enhancing their well-being has been a critical issue (Morrison, 2002), and a sense of calling might be a one way to promote newcomer’s well-being and job performance. HR managers can help newcomers identity and develop a sense of calling by having them a growing awareness of self (Elangovan et al., 2010) or providing calling workshops as a part of training programs (e.g., Dik and Steger, 2008).

Second, the finding that perceived organizational support strengthens the relation between perception of calling and job satisfaction indicates that continued efforts to heighten perceived organizational support can be beneficial for newcomers who view their work as a calling. The perception of being supported and valued by organization partly stems for leader’s supportiveness and perceived leader support and organizational support were found to be compensatory (Maertz et al., 2007). Given that leader’s supervisor support declined during 6**–**21 months after organizational entry and the decline predicted steeper decline in newcomer’s job satisfaction (Jokisaari and Nurmi, 2009), continuous formal and informal programs may help newcomers maintain a certain level of organizational support. Because formal programs can be restricted due to resource constraints, HR managers and leaders could help extend newcomer’s ties by sponsoring him/her how to enhance integration into informal networks that can increase newcomers’ successful socialization (Morrison, 2002).

### Limitations and Directions for Future Research

The results of this study are subject to several limitations that can be addressed in future research. First, our findings may be restricted by the subject sample; whether the results are generalizable to other types of employees or employees in different cultural contexts warrants investigation. Also, cultural differences are present in newcomers’ behaviors and perceptions of organizational tactics (Morrison et al., 2004). Collectivistic cultures such as South Korea are more likely to assign weight to relatedness and organizational support than in America (Kitayama et al., 2000), and this may strengthen the relation between having a calling and perceived organizational support on job involvement. Future research that includes different contexts and diverse types of employees would be valuable.

Second, although the longitudinal nature of the research can alleviate the potential for method variance, all data except for job performance were collected from self-reports, and the study variables were measured only once. Measuring the constructs at more time points and examining reciprocal influences on the variables are necessary to substantiate and clarify the findings in this study. Given that positive views toward work and organization peaked during first few weeks after organizational entry (Boswell et al., 2009), our findings based on calling measured at the very first week of organizational entry should be interpreted with caution and more longitudinal research during the career transition period is needed to strengthen the results of this study.

Finally, although the mediator and moderators in this study were chosen based on prior findings, when and why calling upon organizational entry relates to job performance needs more future research with solid theoretical background. Job performance in this study was rated by a forced distribution approach and the approach has been criticized for rewarding people with high visibility by facilitating political game playing (Guralnik et al., 2004; Schleicher et al., 2009). In a competitive environment that fosters competition and facilitates political behaviors, how people with callings react to the environment and what factors mediate the link between calling and supervisor-rated job performance during the socialization period await future research. In particular, based on a career calling model (Hall and Chandler, 2005; Praskova et al., 2014) and Self-Determination Theory (Deci and Ryan, 2000), what type of goal-directed efforts lead to job performance and how satisfaction of different types of psychological needs influence the effects of calling on job performance would be an interesting area for future research.

## Data Availability

The datasets for this manuscript are not publicly available because our data include objective performance data provided by company. Participants had not been informed that their performance data would be publicly available although they provided consent on the use of their performance data for research purpose. The data are available upon request. Requests to access the datasets should be directed to jyp111@gmail.com.

## Author Contributions

JP, ML, and YS contributed to the conception and design of the study. JP and ML organized the database. JP and SK conducted the statistical analyses. JP wrote the first draft of the manuscript. All authors contributed to the manuscript revision, read, and approved the submitted version of the manuscript.

## Conflict of Interest Statement

The authors declare that the research was conducted in the absence of any commercial or financial relationships that could be construed as a potential conflict of interest.
